# Integrated Phenotypic and Molecular Evaluation of Powdery Mildew Resistance in Egyptian Barley: Identification of Resistance-Associated Markers

**DOI:** 10.3390/plants14081231

**Published:** 2025-04-17

**Authors:** Mariam H. M. El Nabawy, Khadegah M. A. Najeeb, Hala B. Khalil, Khaled A. Soliman, Alia A. El-Seoudy

**Affiliations:** 1 Department of Genetics, Faculty of Agriculture, Ain Shams University, 68 Hadayek Shoubra, Cairo 11241, Egypt; 2 Wheat Diseases Department, Plant Pathology Research Institute, Agriculture Research Center, Giza 12112, Egypt; 3 Biological Sciences Department, College of Science, King Faisal University, P.O. Box 380, Al-Ahsa 31982, Saudi Arabia

**Keywords:** powdery mildew, barley, phenotypic assessments, SSR markers, Egyptian resistance genotypes

## Abstract

Powdery mildew, caused by *Blumeria graminis* f. sp. *hordei* (*Bgh*), severely impacts global barley *Hordeum vulgare* L. (*Hv*) production. This investigation evaluated Egyptian barley genotypes to identify novel resistance sources and molecular markers for breeding programs. Phenotypic assessments at the seedling (growth stage, GS 32) and adult plant (GS 55–59) stages under controlled and field conditions, combined with SSR marker analysis, revealed distinct resistance profiles. Genotypes Giza 123, Giza 125, and G8 exhibited strong resistance, with Giza 123 displaying *Mlo*-mediated immunity. Susceptible genotypes (Giza 126, G1, G2, and G4) showed rapid disease progression (IT4; up to 80% severity). Intermediate genotypes (G5, G6, and G9) suggested quantitative resistance. Simple sequence repeat (SSR) analysis linked the *EBmac0603* primer 160 bp allele to resistance and the 149 bp allele to susceptibility. The *EBmac0603* primer 185 bp allele correlated with partial resistance, highlighting its utility in marker-assisted selection (MAS). The integration of phenotypic and molecular data identified Giza 123 and G8 as prime candidates for breeding, emphasizing the need for strategies like gene pyramiding or quantitative resistance incorporation in susceptible lines. This study underscores the value of Egypt’s barley diversity in advancing durable disease resistance through targeted breeding and molecular tools.

## 1. Introduction

Barley, *Hordeum vulgare* L. (*Hv*), is a vital cereal crop globally, ranking fourth in worldwide production after maize, rice, and wheat [1]. Its resilience and adaptability to harsh environments make it particularly important in arid regions. In Egypt, barley has been an integral part of agriculture for millennia. Today, it remains a significant crop, particularly in marginal lands and areas facing environmental challenges. Despite these challenges, barley continues to be an important crop in Egypt, with its cultivation spread across various regions. However, barley production faces mounting challenges from biotic stresses, particularly powdery mildew caused by *Blumeria graminis* f. sp. *hordei* (*Bgh*), which can reduce yields by up to 40% under severe epidemics [2].

Powdery mildew is a major foliar disease affecting barley, causing significant yield losses. Early infections can reduce yields by up to 25% in susceptible barley varieties, while later infections may lead to losses of around 10% [3]. The disease is widespread in barley-growing regions, particularly in cool and humid climates. This obligate biotrophic fungus colonizes leaf surfaces, forming dense mycelial mats that disrupt photosynthesis and accelerate senescence [4]. Compounding this threat, climate change is altering pathogen dynamics, with warmer temperatures and erratic humidity favoring *Bgh* proliferation [5]. The forma specialis *Bgh*, traditionally classified based on host specificity (barley), has been re-evaluated through genetic analyses of multi-gene DNA sequences. These molecular data support its reclassification as *Blumeria hordei* [6]. Both nomenclatures are included here to reflect ongoing mycological consensus. Developing resistant varieties remains the most effective strategy for managing powdery mildew in sustainable cropping systems, minimizing both economic and labor costs for farmers [3]. Traditional resistance breeding, reliant on major genes such as Mildew Locus O, *Mlo*, has been hampered by the pathogen’s rapid evolution. Virulent *Bgh* isolates frequently overcome race-specific resistance, while the pleiotropic effects of *Mlo* mutations confer broad-spectrum resistance [2,7]. Modern barley breeding strategies are evolving to address the complex challenges of disease resistance and environmental adaptation [8]. These approaches now emphasize gene pyramiding, which involves stacking multiple resistance loci (such as *Mlo* with QTLs like *Ror1* and *Ror2*) to enhance durability while mitigating pleiotropic trade-offs [9]. Advanced techniques, including marker-assisted selection (MAS) and CRISPR-Cas9 genome editing, enable the precise introgression of resistance alleles without linkage drag. These integrated approaches represent a more comprehensive strategy for developing barley varieties that are disease-resistant to the fungus.

Advances in molecular marker technologies, particularly simple sequence repeats (SSRs), provide a pathway to resolve these trade-offs. SSRs, with their high polymorphism and codominant inheritance, enable the precise tracking of resistance alleles and QTLs across diverse germplasm [10]. For instance, markers such as *EBmac0603* and *Bmac0213* have been linked to *Rph23* (leaf rust) and powdery mildew loci, respectively, in global barley collections [11]. However, Egyptian barley genotypes remain underexplored in this context, despite their unique genetic heritage shaped by millennia of adaptation to local stresses.

This study aims to comprehensively evaluate the morphological and molecular characteristics of fifteen Egyptian barley genotypes, focusing on their responses to powdery mildew. By assessing disease severity and employing molecular markers, this research seeks to identify genotypes with enhanced resistance, which can contribute to improving barley production in disease-prone regions. These resistant genotypes could serve as valuable genetic resources for developing new barley varieties with improved disease tolerance, ultimately enhancing crop productivity and sustainability. Additionally, the findings of this study may provide insights for supporting future research on disease management strategies in barley cultivation.

## 2. Results

### 2.1. Differential Symptom Progression in Barley Genotypes Challenged with Powdery Mildew at the Seedling Stage

**Figure 1:**
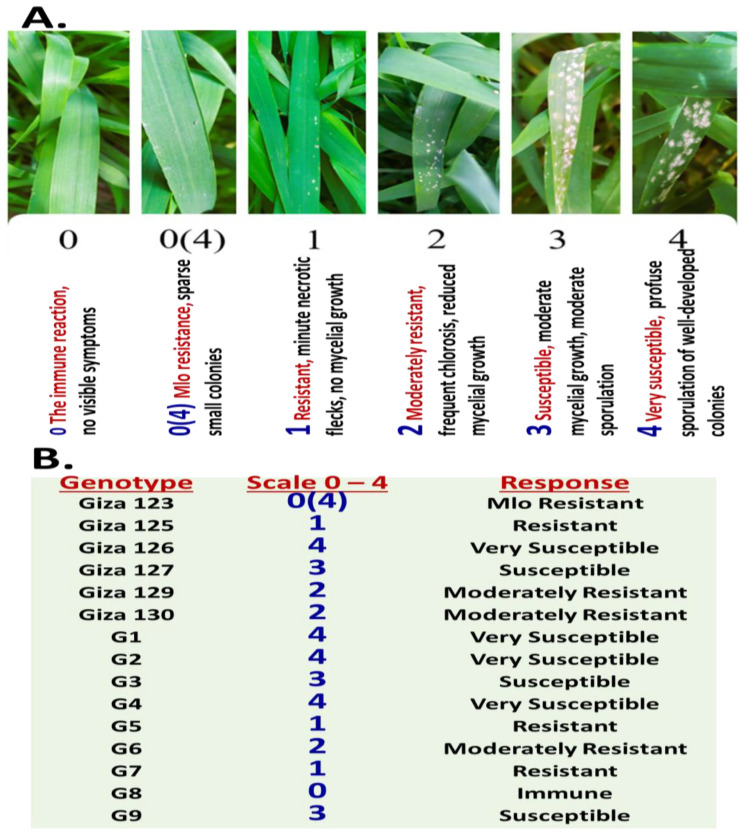
Evaluation of powdery mildew resistance and symptom progression in 15 Egyptian barley genotypes. (**A**) IT scoring scale (IT0–4). Scores IT0–2 denote resistance: IT0 = immune (no signs of infection or tissue response); IT0 (4) = Mildew Locus O, Mlo-mediated resistance (no visible symptoms but microscopic fungal hyphae detected); IT1–2 = partial resistance (small pustules with limited fungal development, and the plant restricts pathogen growth but does not completely prevent it); scores IT3–4 indicate susceptibility: 3 = moderate sporulation (moderate pustule development with visible fungal growth, and disease is clearly established, though not at the most severe level) and 4 = severe infection with abundant sporulation (abundant pustules with heavy fungal growth, severe infection, extensive colonization by the fungus and pronounced disease symptoms). (**B**) Disease development and symptom progression across the 15 genotypes at 7 dpi.

Fifteen Egyptian barley (*Hv*) genotypes at the early seedling stage (growth stage 32, GS 32) were evaluated for resistance to powdery mildew caused by the fungal pathogen Bgh. These genotypes, selected for their diverse agronomic and morphological traits (e.g., row type, growth habit, flag leaf orientation, and pedigree), represented a broad genetic spectrum, including regional accessions and advanced breeding lines (Methods). Resistance assessment was conducted using a standardized 0–4 infection type (IT) scale [12], where scores IT0–2 denoted resistance (IT0 = immune/no symptoms, IT1–2 = partial resistance with restricted fungal growth) and IT3–4 indicated susceptibility (IT3 = moderate sporulation, IT4 = abundant sporulation and tissue damage). A score of IT0 (4) specifically reflected Mlo-mediated resistance, a monogenic trait conferring broad-spectrum immunity through epidermal cell death (Figure 1A). Disease progression was monitored over ten days post-inoculation (dpi). During the initial 2–3 dpi, no visible symptoms were observed across any genotype, likely corresponding to the pathogen’s latent phase. By 4 dpi, genotypes G1 and G2 exhibited mild chlorotic flecks, signaling early susceptibility, while others remained asymptomatic. Full symptom expression occurred by 6–7 dpi in the susceptible genotypes such as Giza 126, G1, G2, and G4 (Figure 1B). Among the fifteen genotypes, three (20%) demonstrated resistance: G8 (immune, IT0), Giza 123 (Mlo-mediated, IT0 (4)), and Giza 125, G5, and G7 (resistant, IT1). Additional genotypes, such as Giza 129, Giza 130, and G6, were classified as IT2 (partial resistance). Susceptibility was divided into two categories: IT3 (moderate susceptibility: Giza 127, G3, G9) and IT4 (high susceptibility: Giza 126, G1, G2, G4). The IT4 group exhibited rapid fungal proliferation and severe tissue damage, suggesting an absence of functional resistance genes. Conversely, IT3 genotypes displayed intermediate susceptibility, potentially harboring residual quantitative resistance factors.

### 2.2. Field-Based Evaluation of Powdery Mildew Resistance in Egyptian Barley Genotypes

To assess the durability of the fungal resistance under field conditions, the 15 Egyptian barley genotypes were monitored weekly for three weeks post-inoculation (wpi) during the critical heading stage (GS 55–59). Disease severity was quantified using an IT0–9 phenotypic scale [13], where IT0 = no symptoms (immune), IT1–3 = partial resistance (trace sporulation), IT4–6 = moderate resistance (partial leaf coverage), and IT7–9 = highly susceptible (extensive fungal colonization and tissue necrosis). The genotypes Giza 123, Giza 125, and G8 exhibited resistance to powdery mildew, displaying minimal infection symptoms (e.g., restricted fungal growth, absence of sporulation) under field conditions. Giza 123, which showed no initial symptoms (score IT0 at 1 wpi), developed mild chlorotic lesions and localized sporulation (score IT3) by 3 wpi, indicative of either delayed susceptibility or incomplete pathogen suppression. In contrast, genotype G8 maintained consistent resistance (fixed score IT1) throughout the assessment period, reflecting stable immunity to Bgh under field conditions.

The genotypes G127, G129, G130, G1, and G2 exhibited susceptible scores (IT5–9) and were classified as highly susceptible to powdery mildew. Progressive susceptibility was observed in genotypes Giza 127 and G1, which transitioned from moderate initial scores (Giza 127: 5 at 1 wpi; G1: IT6 at 1 wpi) to maximal severity (IT9) by 3 wpi, indicating a breakdown of resistance under sustained pathogen pressure (Table 1). Genotypes G2, G4, and G9 exhibited intermediate susceptibility (scores IT5–8), with G4 uniquely showing necrotic flecking alongside sporulation, a possible hypersensitive response delaying fungal spread. Fluctuating responses in G3, G6, and G7 (score variations of ±2–3 between weeks) hinted at the environmental modulation of resistance, potentially influenced by microclimate variability (e.g., dew duration, temperature shifts) or heterogeneous pathogen populations.

**Figure 2:**
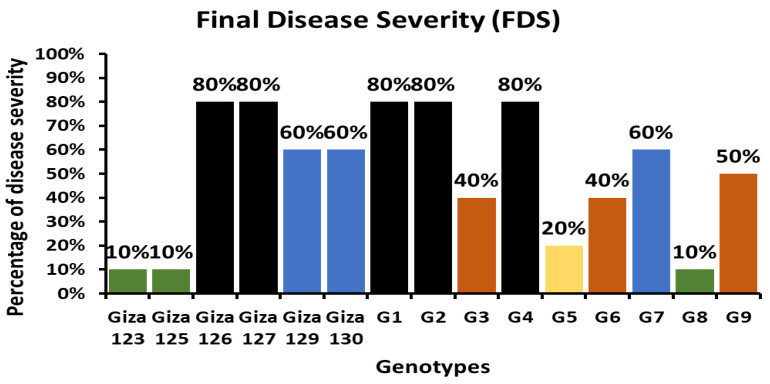
Final powdery mildew disease severity in 15 Egyptian barley genotypes under open-field conditions.

The powdery mildew severity varied markedly among the evaluated barley genotypes (Table 2). Resistance (10–20% severity) was observed in 27% of the genotypes (Giza 123, Giza 125, and G8), while susceptibility dominated in six genotypes (Giza 127, Giza 129, Giza 130, G1, G2, and G4, which revealed the highest severity (80%)). In addition, we categorized the 15 genotypes into five classes based on their final disease severity, including resistant (R: 10–20%), moderately resistant (MR: 20–30%), moderately susceptible (MS: 30–40%), susceptible (S: 50–60%), and highly susceptible (HS: 70–80%). The barley genotypes were categorized into five categories based on their final disease severity (Figure 2), including resistant (R: Giza 123, Giza 125, and G8), moderately resistant (MR: G5), moderately susceptible (MS: G3, G6, and G9), susceptible (S: Giza 129, Giza 130, and G7), and highly susceptible (HS: Giza 126, Giza 127, G1, G2, and G4).

### 2.3. AUDPC-Based Assessment of Powdery Mildew Resistance

The area under the disease progress curve (AUDPC), a robust metric integrating infection intensity and temporal progression, was employed to evaluate powdery mildew resistance across the 15 Egyptian barley genotypes (Table 3). Genotypes Giza 123 (AUDPC = 301), Giza 125 (385), and G8 (385) exhibited the lowest AUDPC values, indicative of durable resistance, while G1, G2, G4 (AUDPC = 3360), and Giza 126 (3290) displayed the highest susceptibility. Apparent infection rates (r-values), derived from disease progression curves, further stratified the resistance dynamics: genotypes G9 (r = 0.0063), G3, and G6 (r = 0.0069) showed the slowest epidemic development, whereas Giza 123 (r = 0.0198), Giza 127 (0.0215), and G7 (0.0278) exhibited accelerated disease spread despite moderate AUDPC scores.

The identification of low-AUDPC genotypes such as Giza 123 and G8 prioritized their use in pyramiding Mlo-independent resistance genes to enhance durable powdery mildew resistance. Additionally, high r-values in partially resistant lines like Giza 127 underscored the risk of resistance erosion under high pathogen pressure, warranting integrated strategies such as intercropping to complement genetic resistance.

### 2.4. Resistance–Susceptibility Gradients to Powdery Mildew in Tested Genotypes

**Figure 3:**
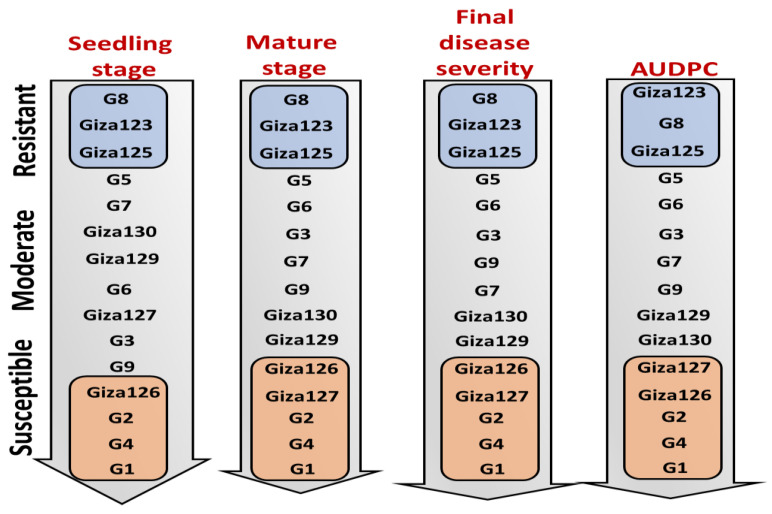
Stratification of barley genotypes along a resistance–susceptibility gradient to powdery mildew. Genotypes were categorized based on disease response at the seedling stage, mature stage, final disease severity, and AUDPC. Resistance and susceptibility levels were determined by integrating phenotypic assessments across these parameters, providing a comprehensive classification of genotypic responses to powdery mildew infection.

The 15 barley genotypes were stratified along a resistance–susceptibility gradient using integrated phenotypic evaluations spanning seedling and adult plant growth stages, coupled with longitudinal disease severity assessments and AUDPC quantification. Resistant genotypes (G8, Giza 123, and Giza 125) exhibited consistent resistance across all stages (Figure 3). In contrast, highly susceptible genotypes (Giza 126, Giza 127, G1, G2, and G4) displayed rapid disease progression, with AUDPC scores exceeding 3290 and severity peaking at 80–90% by maturity. Intermediate genotypes, including G3, G5, G6, G7, G9, Giza 129, and Giza 130, displayed variable responses, with their AUDPC values (840–2240) and severity scores (20–60%) suggesting partial resistance mechanisms.

### 2.5. SSR Unveiled Band-Associated Resistance to Powdery Mildew in Barley Genotypes

**Figure 4:**
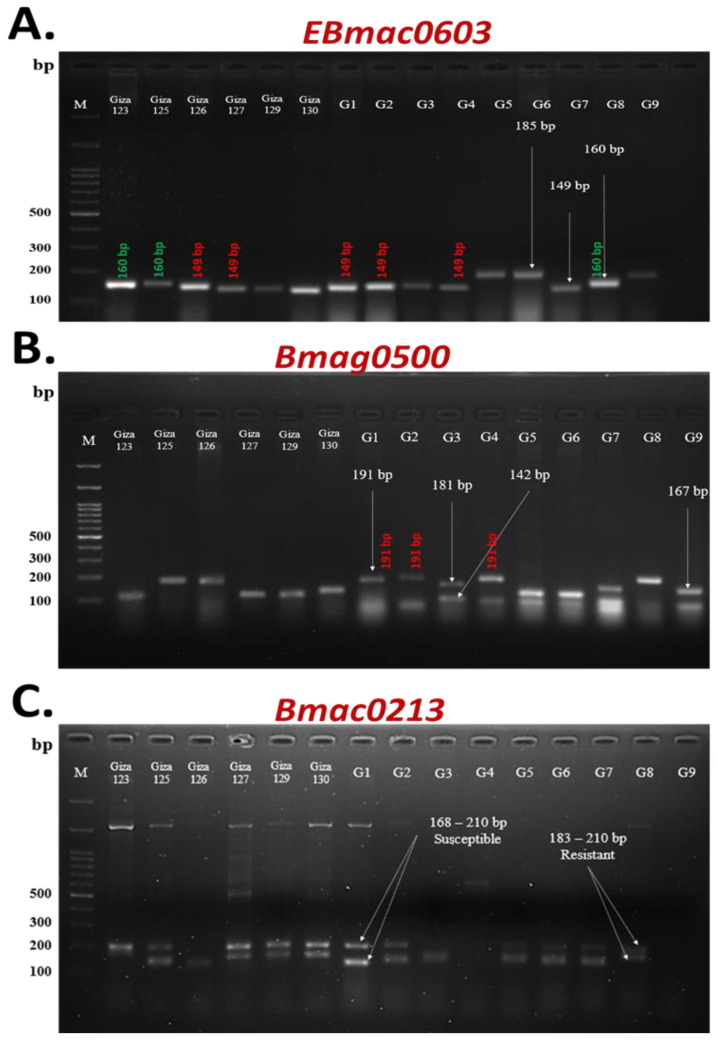
SSR marker profiling of powdery mildew resistance-associated alleles in 15 Egyptian barley genotypes. (**A**) *EBmac0603* primer amplification products resolved via agarose gel electrophoresis, showing three alleles (149, 160, 185 bp) at 100% polymorphism. (**B**) *Bmag0500* primer fragment sizes (142–191 bp) across genotypes. (**C**) *Bmac0213* primer fragment size–resistance correlation: 168–210 bp (susceptible); 183–210 bp (resistant).

Three SSR primers, including *EBmac0603*, *Bmag0500*, and *Bmac0213*, revealed substantial genetic diversity among barley genotypes, each comprising forward and reverse primers targeting three distinct loci. *EBmac0603* displayed three distinct bands at 149, 160, and 185 bp (Figure 4A). The 160 bp band was associated with resistance in the three genotypes (Giza 123, Giza 125, and G8), while the 149 bp band was detected in the five susceptible barley genotypes (Giza 126, Giza 127, G2, G4, and G1). A novel 185 bp allele, found exclusively in three moderately resistant or susceptible genotypes (G5, G6, and G9), emerged as a potential marker for selective breeding. This allele size–resistance pattern highlighted the utility of *EBmac0603* in stratifying barley genotypes by disease response, facilitating the targeted selection of lines with durable resistance (160 bp carriers) or partial resilience (185 bp carriers) for breeding programs.

Primer *Bmag0500* amplified polymorphic DNA fragments (95–191 bp) across the barley genotypes (Figure 4B). Susceptible genotypes G1, G2, and G4 shared a conserved 191 bp allele, suggesting a potential susceptibility-linked locus. Heterozygosity, or multi-locus amplification, was inferred in genotypes exhibiting multiple alleles. Intermediate genotypes Giza 130, G7, and G9 displayed a 167 bp fragment, while Giza 125, Giza 126, and G8 shared a 181 bp band. Genotypes Giza 123 and G3 were uniquely associated with a 142 bp fragment. The smallest amplified fragment (95 bp) was observed in G1, G2, and G9, possibly indicating a truncated or recessive allele. These fragment size polymorphisms highlighted allele-specific associations with resistance–susceptibility gradients, providing actionable markers for breeding programs. The *Bmac0213* primer exhibited significant polymorphism across barley genotypes (Figure 4C), with distinct allele size thresholds correlating to disease response. Amplicons spanning 168–210 bp were strongly associated with susceptible genotypes (e.g., Giza 125, Giza 126, and G1), while a narrower 183–210 bp range tagged the resistant genotypes (Giza 127, Giza 129, Giza 130, G5, G6, G7, and G8).

### 2.6. Integrated Analysis of Egyptian Genotypes

**Figure 5:**
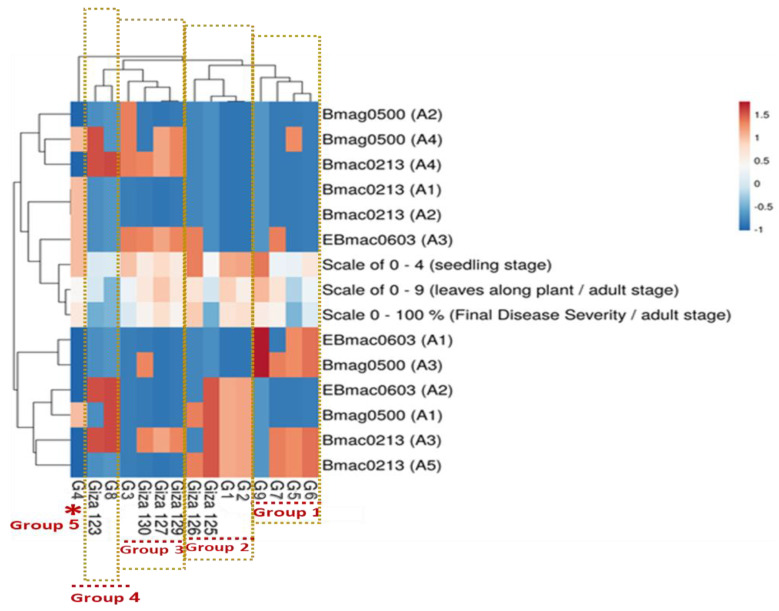
Hierarchical clustering and heatmap analysis of SSR marker associations with powdery mildew resistance in 15 Egyptian barley genotypes. Genotypes were grouped into five distinct clusters based on their resistance levels: Group 1 (G5, G6, G7, and G9) exhibited intermediate resistance, Group 2 (G1, G2, Giza 125, and Giza 126) showed moderate resistance, Group 3 (Giza 123 and G8) contained the most resistant genotypes, Group 4 (Giza 127, Giza 129, Giza 130, and G3) displayed moderate susceptibility, and Group 5 (G4) was the most susceptible. The asterisk (*) near Group 5 (G4) represents the most susceptible genotype and forms a distinct cluster in the molecular analysis.

Hierarchical clustering and heatmap analysis revealed distinct correlations between SSR diversity and phenotypic characteristics across 15 Egyptian barley genotypes, categorizing them into five groups (Figure 5). Group 1 (G5, G6, G7, and G9) comprised genotypes with intermediate resistance, while Group 2 (G1, G2, Giza 125, and Giza 126) exhibited moderate resistance. Group 3 (Giza 123 and G8) contained the most resistant genotypes, displaying minimal disease symptoms. In contrast, Group 4 (Giza 127, Giza 129, Giza 130, and G3) showed moderate susceptibility, with increased disease severity but some level of tolerance, whereas Group 5 (G4) was the most susceptible, exhibiting the highest disease severity. This classification highlighted the genetic diversity of barley responses to powdery mildew and underscored the potential of SSR markers for stratifying resistance levels, providing valuable insights for targeted breeding programs aimed at improving disease resistance.

## 3. Discussion

Powdery mildew, incited by the obligate biotrophic fungus (*Bgh*), poses a significant threat to barley (*Hv*) production worldwide. Deployment of resistant cultivars represents the most effective, economical, and environmentally sound approach to managing powdery mildew. Egyptian barley genotypes, adapted to the environmental conditions of the region, exhibit a diverse range of agronomic traits and genetic backgrounds. This genetic diversity provides a valuable resource for identifying novel sources of resistance and dissecting the underlying genetic mechanisms. Characterizing the resistance profiles of these genotypes from the seedling to adult plant stages is crucial for selecting lines with stable and effective resistance [2]. By integrating phenotypic disease assessments under controlled and field conditions, coupled with molecular marker analysis using SSRs, a comprehensive evaluation of resistance against powdery mildew can be achieved [9]. The ultimate goal is to identify resistant genotypes and their associated molecular markers, which can then be effectively utilized in MAS and breeding programs focused on enhancing disease resistance in barley cultivars.

The differential symptom progression observed among the 15 Egyptian barley genotypes challenged with *Bgh* underscored the complexity of host–pathogen interactions and highlighted the presence of varying resistance mechanisms. Evaluations at the early GS 32 revealed a clear distinction between resistant and susceptible genotypes, allowing for an initial stratification based on IT scores. Notably, Giza 123, Giza 125, and G8 demonstrated strong resistance, exhibiting immunity (IT0) or partial resistance (IT1), characterized by restricted fungal growth and limited sporulation. Conversely, Giza 126, G1, G2, and G4 exhibited rapid disease progression and extensive sporulation, indicative of high susceptibility (IT4) and the absence of effective resistance genes. The resistance observed in Giza 123, particularly its *Mlo*-mediated resistance (IT0 (4)), is of significant interest, as *Mlo* represents a durable, broad-spectrum, monogenic resistance mechanism effective against all known races of Bgh [2,7]. However, *Mlo*-mediated resistance is often associated with undesirable pleiotropic effects, necessitating careful consideration in breeding programs [14]. The intermediate responses observed in genotypes such as G5, G6, and G9 (IT2–IT3) suggested the potential involvement of quantitative resistance (QR) factors, which are typically controlled by multiple genes and provide partial, but often more durable, resistance.

Field evaluations at the mature stage (GS 55–59) were critical for validating the stability and effectiveness of resistance under more realistic and dynamic environmental conditions. The genotypes that exhibited early-stage resistance, particularly Giza 123 and G8, generally maintained their low disease severity scores under field conditions, reinforcing the durability of their resistance. Interestingly, while Giza 123 showed delayed susceptibility at later stages (3 weeks post-inoculation), developing mild chlorotic lesions and localized sporulation (score 3), G8 maintained consistent resistance (score 1) throughout the assessment period. This suggests that G8 possesses additional genetic factors conferring a more robust and long-lasting disease suppression mechanism. In contrast, highly susceptible genotypes such as Giza 126, Giza 127, and G1 demonstrated an increasing disease severity trend, with final scores reaching up to 80–90% on a 0–100% scale, indicating the breakdown of resistance under prolonged pathogen pressure and highlighting their vulnerability to powdery mildew infection.

Molecular analysis using SSR may provide insights into the genetic markers and allow for the identification of specific DNA fragments associated with resistant or susceptible phenotypes. The association of the 160 bp allele amplified by the *EBmac0603* primer with resistant genotypes (Giza 123, Giza 125, and G8) strongly supports its potential as a tightly linked molecular marker for selecting resistant lines in breeding programs. Furthermore, the presence of the 185 bp allele, also amplified by *EBmac0603*, in intermediate-resistant genotypes (G5, G6, and G9) suggests its potential role in conferring partial resistance. The identification of susceptibility-linked alleles, such as the 191 bp fragment amplified by the *Bmag0500* primer in highly susceptible genotypes (G1, G2, and G4), further underscores the utility of SSR markers in MAS and breeding programs aimed at improving disease resistance. These markers can be used to screen large populations of barley seedlings and select individuals carrying the desired resistance alleles, thereby accelerating the breeding process and reducing the need for extensive phenotypic evaluations [15].

The integration of phenotypic, molecular, and field-based assessments provides a comprehensive classification of barley genotypes along a resistance–susceptibility gradient, enabling a more informed selection of parental lines for breeding purposes. Genotypes such as Giza 123 and G8 emerge as particularly promising candidates for breeding programs due to their consistent resistance across multiple evaluation methods and their association with specific resistance-linked SSR markers. In contrast, the high AUDPC values observed in susceptible genotypes highlight the need for alternative management strategies, such as the incorporation of QR genes, the deployment of gene pyramiding strategies, or the judicious use of fungicide applications in highly vulnerable cultivars, to minimize yield losses.

The identification of resistance-associated SSR markers offers a practical tool for MAS, expediting the development of disease-resistant barley varieties. Future research should focus on validating these markers in broader genetic backgrounds, exploring gene expression profiles using techniques such as RNA sequencing to uncover additional resistance pathways against *Bgh*, and fine mapping the genomic regions associated with the identified SSR markers to identify the underlying resistance genes. Furthermore, investigating the interaction between different resistance genes through gene pyramiding could lead to the development of barley cultivars with more durable and effective resistance to powdery mildew.

The association of SSR markers with functional genes, such as pathogen recognition receptors (PRRs), nucleotide-binding leucine-rich repeat (NLR) genes, or other defense-related genes, offers a critical bridge between genetic markers and biological mechanisms in plant–pathogen interactions [16,17]. While SSRs themselves are non-coding repetitive sequences, their physical proximity to functional genes in the genome can result in tight linkage disequilibrium, enabling their use as proxies for tracking the allelic variants of nearby genes. Advances in genome sequencing and comparative mapping can further resolve the question of whether SSRs directly tag functional genes or are part of regulatory haplotypes modulating quantitative resistance.

## 4. Materials and Methods

### 4.1. Experimental Sites

This study was conducted from 2022 to 2024 through a collaborative effort between the Department of Genetics at Ain Shams University and the Agricultural Research Center (ARC). At Ain Shams University, experimental planning, genetic analyses, and data interpretation were coordinated. Field trials and greenhouse experiments were carried out at ARC’s experimental stations, where controlled-environment conditions were maintained for seedling-stage inoculations, while open-field evaluations assessed disease progression.

### 4.2. Plant Materials

The barley genotypes evaluated in this study were obtained from the National Gene Bank (NGB) in Giza, Egypt, to assess their resistance to powdery mildew, *Blumeria graminis* f. sp. *Hordei* (*Bgh*). These genotypes, representing a diverse genetic pool, were systematically screened under controlled and field conditions to quantify stress responses, with the comprehensive results summarized in Table 4.

### 4.3. Seedling Test

The experiment followed the method of Arabi et al. [18], with three replicates of each genotype sown in sterilized peat-moss-filled plastic flats (60 × 40 × 8 cm), each containing ten seeds. Seedlings were maintained in a greenhouse under controlled conditions (20–22 °C day/16–18 °C night, 16 h photoperiod, 85–95% humidity). At the emerging leaves stage (GS 11–12 [19]), flats were transferred to the field for a four-night exposure to natural *Pgh* populations. Symptoms were assessed ten days post-exposure at GS 32, with disease pressure amplified by surrounding spreader rows of susceptible genotypes pre-inoculated with a virulent isolate mixture. This dual-phase approach ensured standardized pathogen challenge and accurate resistance phenotyping.

### 4.4. Seedling-Stage Evaluation of Powdery Mildew Resistance

At the seedling stage (GS 32), ITs were assessed 10 days post-inoculation with *Bgh* using a standardized IT0–4 scale [12]. Resistance was classified as IT0 (immune), IT0 (4) (*Mlo*-mediated resistance; no symptoms), IT1–IT2 (partial resistance; chlorotic flecks, restricted fungal growth), and susceptibility classified as IT3 (moderate sporulation) or IT4 (severe infection with necrosis). This scoring system enabled the precise differentiation of resistance mechanisms, critical for early-stage selection in breeding programs targeting durable powdery mildew resistance.

### 4.5. Maturity Stage Test

Field trials were conducted at the Wheat Diseases Research Department (Egypt) under natural powdery mildew (*Pgh*) pressure, leveraging annual endemic conditions. The experiment followed a randomized complete block design with three replicates, featuring 1 × 1 m plots (1 m spacing between plots) containing five rows (25 cm apart, 50 seeds/row). Fertilization included 50 kg/ha urea (46% N) and 27 kg/ha superphosphate (33% P_2_O_5_), applied pre-sowing and post-tillering. To enhance the disease pressure, powdery-mildew-infected stubble was introduced at the two-leaf stage, and the plots were misted twice daily using a high-pressure sprayer to maintain high humidity [20]. This setup ensured robust pathogen exposure and standardized disease progression for resistance screening.

### 4.6. Evaluation of Maturity Stage Test

Powdery mildew infections were visually evaluated at the mature plant stage (GS 80) using the scale established by Moseman et al. [21]. Genotypes were classified based on infection severity: plants with 0–30% infection were considered tolerant, those with 30–60% infection were rated as moderately susceptible, and those with more than 60% infection were classified as severely affected.

The genotypes were evaluated for their disease severity using a 0–100% symptom scale. Disease severity (DS) was recorded three times at 10-day intervals over two consecutive seasons and expressed as the percentage of the leaf area covered by powdery mildew, following the method described by Peterson et al. [22].

The collected data were used to calculate three key disease parameters: FDS, AUDPC, and the disease progression rate (r-value). FDS was determined based on the disease severity (%) at the point when the highly susceptible check variety exhibited severe rust symptoms and reached its maximum level of leaf rust severity, as described by Das et al. [23]. AUDPC is a metric used to quantify the cumulative severity of the disease over time, enabling comparisons between genotypes. This calculation was taken at regular intervals. The AUDPC was calculated following the equation provided by Pandey et al. [24], which allowed for the comparison of different genotypic responses to powdery mildew:

(1) $$AUDPC=D\left[\frac{1}{2}\left(Y_{1}+Y_{k}\right)+Y_{2}+Y_{3}+\cdots +Y_{\left(K-1\right)}\right]$$

D: constant time interval (days) between consecutive disease assessments; Y_1_: disease severity (%) at the first observation; Y_k_: disease severity (%) at the final observation; and Y_2_, Y_3_, …, and Y_k−1_: intermediate disease severity readings.

AUDPC integrates both disease severity and progression rate over time, making it a robust metric for comparing resistance across genotypes.

To assess the genotypes’ influence on the progression of leaf disease infection in the field, the rate of disease development (r-value) was calculated based on the disease severity recorded at the disease onset and at 14-day intervals thereafter. The r-value, representing the rate of increase in leaf disease, was determined using the equation proposed by Van der Plank [25]:

(2) $$r\text{-}value=\frac{1}{t_{2}-t_{1}}(\log\frac{X_{2}}{1-X_{2}}-\log\frac{X_{1}}{1-X_{1}})$$

X_1_: the proportion of the susceptible infected tissue (disease severity) at date t_1_; X_2_: the proportion of the susceptible infected tissue (disease severity) at date t_2_; and t_2_ − t_1_: the interval in days between these dates.

This approach allowed for a comprehensive evaluation of disease progression and genotype resistance to powdery mildew in field conditions.

### 4.7. Hierarchical Clustering and Heatmap Visualization

Hierarchical clustering and heatmap visualization were carried out using ClustVis (https://biit.cs.ut.ee/clustvis/; accessed on 1 April 2024), a user-friendly, web-based tool designed for the visualization of multivariate data through principal component analysis and heatmaps [26].

### 4.8. Genomic DNA Extraction

Ten seeds per genotype were surface-sterilized and germinated in sterile Petri dishes under controlled conditions. Leaf tissue was harvested from two-week-old seedlings, flash-frozen in liquid nitrogen, and homogenized. Genomic DNA was extracted using the DNeasy™ Plant Mini Kit (Qiagen Inc., Cat. No. 69104; Hilden, Germany) following the manufacturer’s protocol. This included lysis with AP1 buffer, RNAse A treatment, protein precipitation, and DNA binding/elution in AE buffer. DNA concentration and purity (A260/A280 ratio 1.8–2.0) were quantified via spectrophotometry (NanoDrop™, Thermo Fisher Scientific, Waltham, MA, USA), with integrity verified by 1% agarose gel electrophoresis. Extracted DNA was stored at −80 °C for downstream SSR marker analysis, ensuring consistency across genotyping assays.

### 4.9. SSR-PCR

PCR amplifications were performed in a 20 µL reaction volume using OnePCR™ Ultra (GeneDireX, Inc., Taoyuan, Taiwan). Each reaction contained 10 µL of OnePCR™ Ultra, which included Taq buffer, MgCl_2_, dNTPs, and Taq polymerase [27]. Genomic DNA concentrations for all samples were normalized to 20 ng/μL for each reaction. PCR was carried out using a BOECO Gradient Thermal Cycler (Model: BOE 8089602). Three SSR primers (*EBmac0603*, *Bmag0500*, and *Bmac0213*) were used to assess resistance to powdery mildew. The primers for these markers were selected based on various references, and their sequences are listed in Table 5. DNA integrity was assessed by electrophoresis on 1% (*w*/*v*) agarose gel. PCR amplification products were separated on 1% agarose gel using 1× TBE buffer at 100 V for 2 h, alongside an appropriate DNA ladder for size estimation. Simple sequence repeat (SSR) analysis was based on the presence or absence of alleles, as band intensity is not considered informative for SSR marker interpretation. SSR allele sizes were determined by analyzing the migration patterns of electrophoretic bands using GelAnalyzer (version 23.1.1).

## 5. Conclusions

Overall, this investigation underscores the importance of combining phenotypic and molecular approaches to dissect resistance in barley and to identify valuable sources of resistance for breeding programs. By integrating multi-stage phenotypic evaluations and molecular marker analysis, we identified genotypes with distinct resistance mechanisms. Giza 123 and G8 emerged as key candidates due to their robust resistance at both the seedling and adult plant stages, with G8 demonstrating exceptional durability under field conditions. While Giza 123′s *Mlo*-mediated immunity offers broad-spectrum resistance, its association with pleiotropic trade-offs necessitates careful deployment, underscoring the importance of balancing qualitative and quantitative resistance strategies. Molecular marker analysis revealed that SSR alleles can streamline MAS for resistance. These findings advocate for gene pyramiding approaches and for the integration of genomic tools to optimize resistance breeding. Overall, this work underscores the importance of leveraging regionally adapted genetic diversity to develop climate-resilient barley cultivars. Future efforts should focus on field validations of resistance stability, the functional characterization of candidate genes, and breeding programs that synergize Egypt’s genetic resources with modern genomic technologies to mitigate powdery mildew threats sustainably.

## Figures and Tables

**Table 1:** Progression of powdery mildew severity (IT0–9 scale) in 15 Egyptian barley genotypes at maturity stage under field conditions.

Genotypes	Scale of IT0–9 (Leaves Along Plant)		
	1st Read	2nd Read	3rd Read
Giza 123	0	1	3
Giza 125	1	1	3
Giza 126	5	7	7
Giza 127	5	7	9
Giza 129	5	5	7
Giza 130	5	5	7
G1	7	7	9
G2	7	7	7
G3	3	5	5
G4	7	7	7
G5	3	3	3
G6	3	5	5
G7	3	4	7
G8	1	1	1
G9	3	7	7

IT0 (dark blue): no visible infection; IT1 (medium-dark blue): minor flecks or hypersensitive; IT2 (medium blue): small uredinia with necrosis; IT3 (light blue): Moderate sporulation with chlorosis; IT4 (very light blue): moderate to heavy sporulation; IT5: transitional score; IT6 (light red): noticeable sporulation; IT7 (medium red), IT8 (darker red), and IT9 (dark red): maximum susceptibility with heavy sporulation and no resistance response.

**Table 2:** Final disease severity (0–100% scale) of powdery mildew infection in 15 Egyptian barley genotypes at mature stage, classified by resistance categories.

Genotypes	Final Disease Severity (0–100%)		
	1st Read	2nd Read	3rd Read
Giza 123	0%	5%	10%
Giza 125	5%	10%	10%
Giza 126	70%	80%	80%
Giza 127	50%	70%	80%
Giza 129	40%	50%	60%
Giza 130	40%	50%	60%
G1	70%	80%	80%
G2	70%	80%	80%
G3	30%	40%	40%
G4	70%	80%	80%
G5	10%	20%	20%
G6	30%	40%	40%
G7	20%	40%	60%
G8	5%	10%	10%
G9	40%	50%	50%

**Table 3:** Area under the disease progress curve (AUDPC) and apparent infection rate (r) for determining powdery mildew severity in 15 Egyptian barley genotypes.

Genotypes	AUDPC	r-Value
Giza 123	301	0.0198
Giza 125	385	0.0116
Giza 126	3290	0.0084
Giza 127	3010	0.0215
Giza 129	2240	0.0126
Giza 130	2240	0.0126
G1	3360	0.0084
G2	3360	0.0084
G3	1680	0.0069
G4	3360	0.0084
G5	840	0.0126
G6	1610	0.0069
G7	1960	0.0278
G8	385	0.0116
G9	2030	0.0063

**Table 4:** Agronomic traits, growth habits, and pedigree details of the fifteen Egyptian barley genotypes.

Genotype	Row Type	Growth Habit	Flag Leaf	Pedigree
Giza 123	Six	Semi-prostrate	Horizontal	Giza 117/FAO 86
Giza 125	Six	Semi-prostrate	Semi-drooping	Giza 117/Bahteem52//Giza118/FAO86 (sister line to Giza 124)
Giza 126	Six	Semi-prostrate	Horizontal	Baladi Bahteem/S D729-Por12762-BC
Giza 127	Two	Intermediate	Semi-erect	W12291/B0gs//Hamal-02
Giza 129	Six	Semi-prostrate	Semi-drooping	Deir Alla 106/Cel//As46/Aths * 2’’ Comp.cross”
Giza 130	Six	Intermediate	Semi-erect	229//Bco.Mr./DZ02391/3/Deir Alla 106 CM67B/CENTENO//CAMB/3/ROW906.73/4/GLORIABAR/COME-B/5/
G1	Six	Intermediate	Semi-erect	El Minya (Egy., GenBank, code No. 11331)
G2	Six	Intermediate	Horizontal	El Minya (Egy., GenBank, code No. 11333)
G3	Six	Intermediate	Semi-erect	El Minya (Egy., GenBank, code No. 11337)
G4	Six	Semi-erect	Semi-erect	El Minya (Egy., GenBank, code No. 11338)
G5	Six	Semi-erect	Erect	El Minya (Egy., GenBank, code No. 11342)
G6	Six	Semi-prostrate	Semi-drooping	Alexandria (Egy., GenBank, code No. 11343)
G7	Six	Intermediate	Horizontal	El Wadi El Gadid, El Dakhla, Mut Agricultural School
G8	Two	Intermediate	Semi-erect	Kafr El-Sheikh—Muhammad Issa El Nataq
G9	Six	Semi-prostrate	Semi-drooping	Kafr El-Sheikh—Wanis Nazih—Al-Rawda

**Table 5:** List of SSR primers used for disease resistance screening in barley genotypes, including their sequences and references.

Primer	Sequences (5′ to 3′)	References
EBmac0603-F	ACCGAAACTAAATGAACTACTTCG	Elakhdar et al. [28]
EBmac0603-R	TGCAAACTGTGCTATTAAGGG	
Bmag0500-F	GGGAACTTGCTAATGAAGAG	Marzougui et al. [29]
Bmag0500-R	AATGTAAGGGAGTGTCCATAG	
Bmac0213-F	ATGGATGCAAGACCAAAC	Yumurtaci [30]
Bmac0213-R	CTATGAGAGGTAGAGCAGCC	

## Data Availability

Data is contained within the article.
